# Cloud Computing-Based Framework for Breast Tumor Image Classification Using Fusion of AlexNet and GLCM Texture Features with Ensemble Multi-Kernel Support Vector Machine (MK-SVM)

**DOI:** 10.1155/2022/7403302

**Published:** 2022-08-31

**Authors:** Jaber Alyami, Tariq Sadad, Amjad Rehman, Fahad Almutairi, Tanzila Saba, Saeed Ali Bahaj, Alhassan Alkhurim

**Affiliations:** ^1^Department of Diagnostic Radiology, Faculty of Applied Medical Sciences, King Abdulaziz University, Jeddah 21589, Saudi Arabia; ^2^Smart Medical Imaging Research Group, King Abdulaziz University, Jeddah 21589, Saudi Arabia; ^3^Animal House Unit, King Fahd Medical Research Center, King Abdulaziz University, Jeddah 21589, Saudi Arabia; ^4^Department of Computer Science & Software Engineering, International Islamic University Islamabad, Islamabad, Pakistan; ^5^Artificial Intelligence & Data Analytics Lab CCIS Prince Sultan University, Riyadh, Saudi Arabia; ^6^MIS Department, College of Business Administration, Prince Sattam Bin Abdulaziz University, Alkharj 11942, Saudi Arabia

## Abstract

Breast cancer is common among women all over the world. Early identification of breast cancer lowers death rates. However, it is difficult to determine whether these are cancerous or noncancerous lesions due to their inconsistencies in image appearance. Machine learning techniques are widely employed in imaging analysis as a diagnostic method for breast cancer classification. However, patients cannot take advantage of remote areas as these systems are unavailable on clouds. Thus, breast cancer detection for remote patients is indispensable, which can only be possible through cloud computing. The user is allowed to feed images into the cloud system, which is further investigated through the computer aided diagnosis (CAD) system. Such systems could also be used to track patients, older adults, especially with disabilities, particularly in remote areas of developing countries that do not have medical facilities and paramedic staff. In the proposed CAD system, a fusion of AlexNet architecture and GLCM (gray-level cooccurrence matrix) features are used to extract distinguishable texture features from breast tissues. Finally, to attain higher precision, an ensemble of MK-SVM is used. For testing purposes, the proposed model is applied to the MIAS dataset, a commonly used breast image database, and achieved 96.26% accuracy.

## 1. Introduction

Breast cancer (BC) is a leading cause of demise for females universally. The WHO stated that expected cancer cases will rise to 19.3 million in 2025 [1]. Several imaging modalities are used to diagnose BC, such as mammography, breast ultrasound, MRI, and computed tomography (CT). Furthermore, microscopic images are also used to find breast cancer [2, 3]. However, mammography is presently one of the recommended diagnostic procedures to detect early-stage breast cancer [4]. The diagnostic procedure called magnetic resonance imaging (MRI) is the mainly suggested substitute for a mammogram.

**Figure 1:**
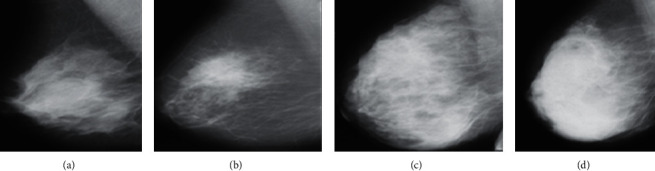
(a) Mass (mdb001), (b) asymmetry (mdb081), (c) calcification (mdb239), and (d) architectural distortion (mdb171).

Nevertheless, the MRI procedure is performed after the existence of the lesion, and radiologists want to verify. The MRI's disadvantage is that it might create a skin infection, allergic reaction, or cause claustrophobia. Three common symptoms of breast cancer are masses, microcalcifications (MCs), and architectural distortion, as presented in Figure 1. There are some other breast cancer signs, but these are not considered. A harmful mass is termed a malignant tumor, whereas a harmless tumor is called benign. The benign tumors are circular, oval, and round shapes, while malignant tumors have irregular boundaries. Furthermore, the malignant tumors look whiter than the surrounding tissue [5].

Over the last few years, cloud computing applications have received considerable attention due to its lower acquisition costs. It includes an online application for IT staff to access all their computing resources remotely and allows data to be integrated into the cloud [6]. In addition, cloud computing offers an ample supply of tools to store and process extensive medical images of big databases [7].

## 2. Literature Review

A breast cancer diagnosis is still a fresh research area and is a field of interest for many researchers [8, 9]. Rodriguez-Ruiz et al. [10] studied an AI system's ability to replace doctors in breast cancer diagnosis. Their findings showed that the AI systems were highly effective than the radiologists in detecting breast cancer. Mughal et al. [11] identified breast cancer using the GLCM and Hat transform to derive features from mammograms. They used the F-test to determine the best features and fed them to BPNN to classify breast images from the MIAS and DDSM datasets. MIAS recorded 95% accuracy for benign-malignant and 98.5% accuracy for normal-abnormal, while DDSM datasets claimed 98% and 99% accuracy. Gupta et al. [12] suggest an artificial cloud-computing model to predict heart disease using the Cleveland dataset. Various algorithms were tested such as random forest, J48, Naïve Bayes, binary discriminant, AdaBoost, and SVM. The AdaBoost classifier performed best on the WDBC dataset at 98.24% accuracy [13] and used cascading of the Fuzzy C-Means (FCM) and region-growing (RG) algorithm to segment tumor in mammograms. Local Binary Pattern Gray-Level Co-occurrence Matrix (LBP-GLCM) and Local Phase Quantization were used to extract features (LPQ). The best features are chosen using the mRMR algorithm. The classifiers are checked on 109 and 72 images of these two databases using k-fold cross-validation. The MIAS dataset has an improved classification accuracy of 98.2%. Using the KNN classifier on LPQ attributes, 95.8% accuracy was achieved for the DDSM dataset.

Vijayarajeswari et al. [14], by using the Hough transform and SVM, achieved an accuracy of 94% on a limited mammogram dataset. Rodriguez-Ruiz et al. [10] proposed a Deep Belief Network using genetic algorithms to fine-tune the network weights and biases. Lastly, the Deep Belief Network fused with an extreme learning machine and claimed 99.99% and 99.12% accuracy using Breast Cancer Wisconsin original (WBCO) and WDBC datasets. Saba et al. [15] addressed the application of cytology images to breast cancer detection and classification using Naive Bayesian and the Artificial Neural Network. They claimed 98% accuracy on breast cytology images. Ragab et al. [16] proposed the CAD system, composed of two components: first, to identify the region of interest and second, to extract features using DCNN. Finally, with support vector machine, an accuracy 87.2% was achieved for predicting breast cancer from mammograms. Ting et al. [17] suggested that such an algorithm could accurately diagnose and identify breast cancer on mammogram images at 90.5% of precision and 90.7% of specificity.

Abdar et al. [18] proposed a voting and stacking technique to create a two-layer one-class ensemble model for BC classification. They achieved 98.07% of accuracy on the WDBC dataset. Assiri et al. [19] used a combination of regression learning, SVM, and MLP to classify mammograms. Their approach reached 99.42% of accuracy on the WBCD dataset. Saba et al., [20] detected benign and malignant tumors using two pretrained DCNN models (AlexNet and DenseNet201) on BUS images. 92.8% of classification accuracy was claimed using the DensNet201 model. Mohiyuddin et al. [21] proposed YOLOv5 to identify and categorize breast cancers on the Curated Breast Imaging Subset of DDSM (CBIS-DDSM). Subsequent to preprocessing, authors claimed 96% of mAP, 93.50% of the MCC value, 96.50% of accuracy, 0.04 of FPR, and 0.03 of the FNR value. It was also asserted that their model outperforms RCNN and YOLOv3 in tests.

The pretrained xception and deeplabv3+ design semantic model was presented by Amin et al. [22]. The segmentation of ultrasound breast images into benign or malignant tissue claimed accuracy of above 95% via tuning of the model's parameters. To identify breast cancer, the segmented images and histological breast images are sent to a 4-qubit quantum circuit with a six-layered design. From the current literature reviewed, it could be seen that most of the systems for breast cancer diagnosis are offline and cannot help remote area patients. Hence, the primary contributions of this study are detailed as follows:

- A cloud-based diagnosis framework is proposed for breast cancer diagnosis of remote areas' patient data.
- From mammogram images, the fused feature vector was developed by extracting handcrafted, deep features through GLCM methodology and AlexNet architecture, respectively.
- Various kernels are ensembled using SVM through majority voting to precisely classify the breast images into normal, benign, and malignant images.

The further research is organized into four main sections: Section 3 presents the in-depth structure of deep convolutional neural networks, Section 4 presents the proposed research methodology, and Section 5 exhibits results and discussion. Finally, Section 6 concludes the research.

## 3. Deep Convolutional Neural Networks (DCNNs)

The idea of DCNNs depends upon the fact that these networks signify the advancement in many image-recognition situations [23]. Furthermore, we want to utilize the essential capability of CNNs to extract features automatically with increasing meaning [24,25]. The state of the art presents different CNNs models, CiFarNet [26], AlexNet [27], GoogLeNet [28], ResNet [29], VGG16, and VGG 19 [30]. Most researchers employed these CNN-based models through transfer learning approach, in which models are trained through the ImageNet dataset [31]. However, we employed AlexNet architecture to achieve deep features, which were further combined with GLCM (gray-level co-occurrence matrix) features. Finally, an ensemble of the multi-kernel SVM is applied to process the fused features vector for classification.

This research has the following main contributions:

- Feature extraction is through the GLCM feature and AlexNet architecture for deep features.
- Fusion of features is done to achieve high accuracy
- Ensemble of the multi-kernel SVM is applied to process fusion features

## 4. Proposed CAD System

The proposed model for breast cancer classification comprises of the following four stages:

- Stage-1 image acquisition
- Stage-2 GLCM and AlexNet features' extraction
- Stage-3 fusion of AlexNet and GLCM features.
- Stage-4 SVM-based classification using ensemble of multi-kernels with majority voting.

**Figure 2:**
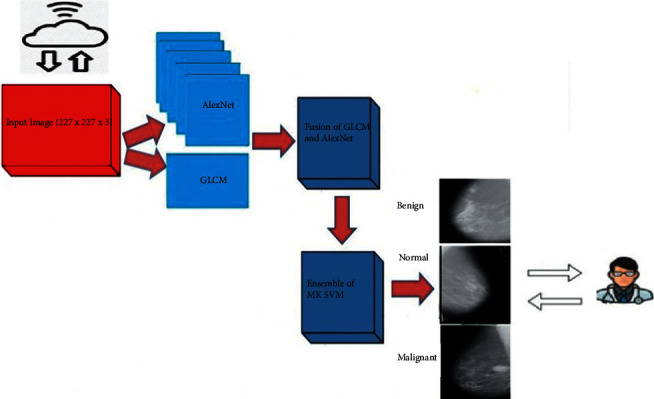
Graphical abstract of the proposed CAD system.

The detailed implementation is provided in the following sections, and the overall design is depicted in the form of graphical abstract in Figure 2.

### 4.1. Image Acquisition

The MIAS (Mammographic Image Analysis Society) database is available publicly [32]. For experiments and to evaluate the proposed approach, we employed 321 mammogram images (206 normal, 63 benign, 52 malignant).

### 4.2. Feature Extraction

#### 4.2.1. Grey Level Co-Occurrence Matrix (GLCM) Features

**Figure 3:**
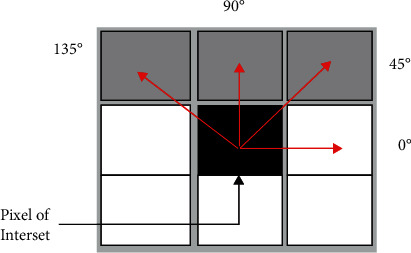
GLCM illustration.

GLCM is utilized for textural features [33]. It provides a detailed interpretation of the image. It calculates the dependency of two brightness values in an image. The calculation of GLCM is a two-step process; formation of the cooccurrence matrix and computation of the texture features. First, the analysis of GLCM among two neighboring values is calculated with the help of displacement *d*=1 and angles *θ*=(0^0^, 45^0^, 90^0^, 135^0^) as illustrated in Figure 3. Subsequently, the cooccurrence matrix is used to extract various statistical attributes, whose details could be found in the study of Sadad et al. [34].

#### 4.2.2. AlexNet Architecture

**Figure 4:**
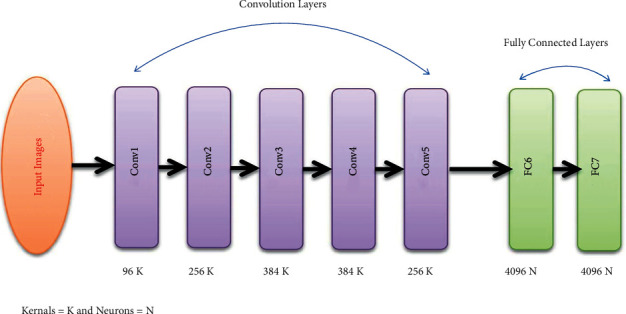
AlexNet architecture for feature extraction.

AlexNet architecture is a variation CNN model [27]. In the proposed AlexNet model, we used five layers of convolutions containing CONV1, CONV2, CONV3, CONV4, and CONV5 and two fully connected (FC) layers, namely, (FC6 and FC7). This method is applied after converting the MIAS dataset from two dimensions into three dimensions because MIAS images are available in the form of two dimensions, which are not according to AlexNet architecture. Thus, images are transformed into three dimensions before being input to CNNs layers. Moreover, the AlexNet model also consists of an FC8 layer, but it has only 1024-dimensional features. Therefore, we consider FC6 and FC7 layers for the extraction of 4096 features from each image of the MIAS database fed to AlexNet architecture. The number of extracted features determined automatically based on the experiments and the highest accuracy are in view.  Figure 4 exhibits proposed AlexNet architecture.

#### 4.2.3. Fusion of Features

Only one type of feature extraction method may limit the object's interpretation capability to classification performance [35]. However, this feature fusion comes out with a distinct descriptor for lesion classification. Therefore, we concatenated GLCM and AlexNet features in the proposed method before processing the classification stage. Thus, we obtained fusion features named Fusion_(Alexnet,GLCM)_ as presented in equation (1). As a result, a total of 5016-dimensional features were finalized, as shown in Table 1.

(1) $$\begin{array}{c} {\text{Fusion}}_{\left(\text{Alexnet,GLCM}\right)}=\text{Conc}\left[{\text{Feature}}_{\text{AlexNet}},{\text{Feature}}_{\text{GLCM}}\right]. \end{array}$$

### 4.3. Classification with Ensemble MK SVM

Following feature extraction and fusion, classification is performed to classify the benign, normal, and malignant ones. Several methods are used to improve the performance of textural features, and among them support vector machine (SVM) is frequently employed. SVM is also useful in multiclassification problems. In SVM, each feature element becomes the value of a particular coordinate. Finally, classification is performed by properly finding the hyperplane that distinguishes the two classes.

As presented in equation (2), SVM mathematically separates the classes through a hyperplane.

(2) $$\begin{array}{c} Y=W*\;\Psi \left(x\right)+b. \end{array}$$

In equation (2), Ψ(*x*) express the nonlinear transformation, where the main focus is on estimating the suitable values of weight and bias called *W* and *b*, respectively. Finally, the regression risk of *Y* is calculated by using the following equation:

(3) $$\begin{array}{c} \text{Reg}\left(Y\right)=C*\sum_{j=0}^{n}\left(Y_{i}-Y_{i}^{\prime }\right)+\frac{1}{2}*W^{2}, \end{array}$$

where *C* and *ϵ* present penalty factor and cost function, respectively. The following equation is used to calculate the weight value:

(4) $$\begin{array}{c} W=\sum_{i=1}^{n}\left(\alpha_{i}-\alpha_{i}^{*}\right)\Psi \left(x_{i}\right). \end{array}$$

In equation (4), the elements *α* and *α*^*∗*^ express a relaxation factor, that are usually known as Lagrange multipliers, which always choose nonzero values. The SVM can be calculated using the following equation:

(5) $$\begin{array}{c} S=\sum_{i=1}^{n}\left(\alpha_{i}-\alpha_{i}^{*}\right)\Psi \left(x_{i}\right)*\Psi \left(x\right)+b,\\ S=\sum_{i=1}^{n}\left(\alpha_{i}-\alpha_{i}^{*}\right)*\kappa \left(x_{i},\;x\right)+b, \end{array}$$

where *κ*(*x*_*i*_,  *x*) denotes the kernel function.

As it is difficult to find a suitable kernel during the learning process in the SVM, thus ensemble various kernel functions are employed in the proposed method [36] The ensemble-SVM is a powerful classification method when various kernels of SVM are ensembled. The most important kernel functions such as SVM-Linear, SVM-Polynomial, SVM-RBF, and SVM-Sigmoid are employed as base classifiers, and selections are made based on the majority vote.

We evaluated the proposed method using 10-fold cross-validation criteria [37]. The outcome is produced in the form of true positive (TP), true negative (TN), false positive (FP), and false negative (FN) to calculate accuracy, confusion matrix, and other statistical results.

**Figure 5:**
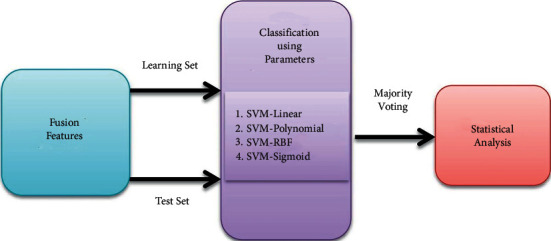
Classification system.

The proposed method classification classifier based on majority voting is shown in Figure 5.

The proposed model architecture is further explained using the following Algorithm 1.

### 4.4. The Prototype Application

**Figure 6:**
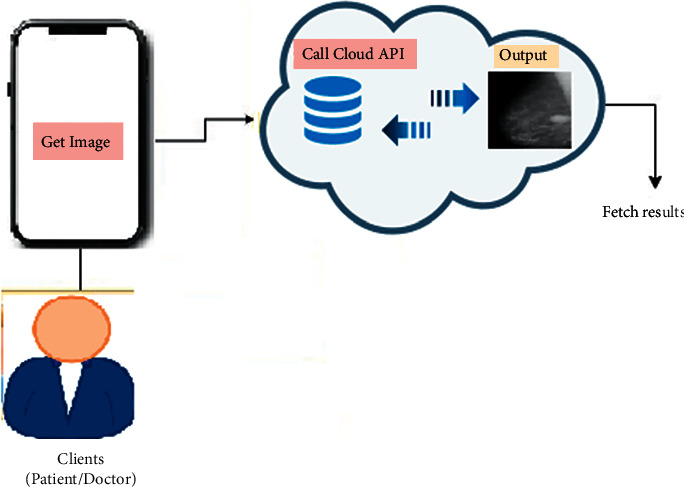
Application architecture.

An integrated cloud application has been developed for breast cancer classification using mammography images as shown in Figure 6. The proposed system allows users to upload an image to feed it to the cloud. The application in the cloud will evaluate the mammogram image and provide the output in the form of normal, benign, or malignant.

## 5. Simulation and Results

**Figure 7:**
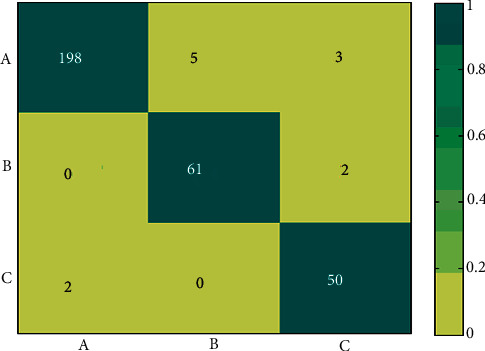
Confusion matrix: (a) normal, (b) benign, and (c) malignant.

This section describes the database and performance measures employed in this research. For experimental purposes, we used 321 images of the MIAS dataset. The images are classified into three classes such as normal, benign, and malignant cases. Fusion features are employed to evaluate the model for breast tumor classification. The experimental results of the MIAS dataset are concisely examined based on the classification performance. The confusion matrix of the proposed method is presented in Figure 7 and other statistical results in Table 2. The classification results are revealed to demonstrate the supremacy of the proposed model.

### 5.1. Analysis and Discussions

This research deliberated a few key points to design a novel kind of hybrid-deep feature through the machine learning model for breast cancer classification; first, most of the researchers used shallow features to achieve classification; second, recent classification based on deep learning has specified that classification accuracy is directly associated with “deep-features.” Therefore, we designed a fusion of shallow and deep features from two different well-known methods to achieve breast cancer classification. Thus, fusion features are utilized to produce robust and powerful features for accurately identifying breast lesions in the proposed CAD system. Moreover, ensemble kernel functions enable a combination of different kernels that are employed. The reason behind selecting multiple kernels in the ensemble method is to merge and get a better classifier result. The MK SVM achieves better results with an ensemble of four Kernel functions. The presented model exhibited 96.2% accuracy, high precision, recall, and *F*1 score for breast tumor classification into normal, benign, and malignant as shown in Table 2. Based on result analysis, it is stated that the highest value of accuracy (96.2%), precision (94%), recall (96%), and *F*1 score (95%) is accomplished with AlexNet + GLCM features. This result also indicates that the proposed features with multi-kernel SVM ensemble is highly efficient for breast cancer classification and allied diagnosis.

### 5.2. Analysis and Comparisons

The efficiency of the proposed model is compared to the state of the art breast cancer classification methods on the MIAS dataset.  Table 3 presents results compared in the state of the art. The methods are compared according to accuracy, indicating the proposed method's supremacy. Deep learning and machine learning both have trade-offs. So, we used their benefits and avoided their drawbacks. In order to get better outcomes, we used both conventional and deep features in the suggested approach of feature fusion.

## 6. Conclusion and Future Direction

In the proposed system, we used the fusion method for feature extraction using mammography images. We extracted 4096 features using AlexNet and 20 features using the GLCM method. Consequently, the feature vector is composed through the fusion of textural features and deep features. Finally, an ensemble of MK-SVM classifiers is utilized for classification. The whole process is carried out on the cloud for cross-validation from experts.

Moreover, the patients from remote areas will be capable to input radiology images into the cloud system, which is further investigated through the CAD system located on the cloud. The proposed method has shown their significant ability to enhance accuracy and achieve remarkable performance on the classification task. The proposed model might inspire a new way to improve the performance of CNNs on specific diagnostic imaging. We will enhance the learning method by employing more and advanced machine learning classifiers in the future. Furthermore, the kernel function of the SVM classifier could [42] be improved [13] further through other ensemble methods. However, further intensive experiments are required for analysis and comparisons. [43].

The main limitation of this research is classification of pattern (cancer) without the feature selection process, which is an important step for dimensionality reduction to enhance machine learning performance. This limitation can be considered in the future work for further enhancement.

## Figures and Tables

**Algorithm 1:**
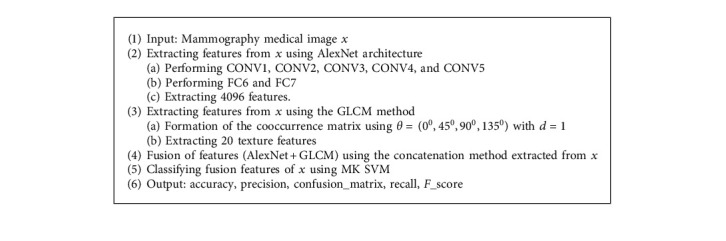
Algorithm 1 Proposed process.

**Table 1:** Fusion feature description.

Features	Size	Description
GLCM	1 × 20	Feature generation based on the second-order method
AlexNet	1 × 4096	Produce deep features

**Table 2:** Performance based on fusion features.

MIAS dataset	Statistic	Value (%)
Classification of normal, benign, and malignant tumors using fusion features	Accuracy	96.2
	Precision	94
	Recall	96
	*F*1 score	95

**Table 3:** Comparison in the state of the art.

References	Methods	Accuracy (%)
Proposed	AlexNet + GLCM + MK SVM	96.2
Amin et al. [22]	Xception and Deeplabv3+	95+
Saba et al. [20]	AlexNet and DenseNet201	92.8
Mohiyuddin et al. [21]	YOLOv5	96.5
Darweesh et al. [38]	LBP + random forest	85
Yu et al. [39]	VGG16	89.06
Shi et al. [40]	CNN	83.6
Saba et al. [15]	Naive Bayesian and artificial neural network	98
Saraswathi et al. [41]	Swarm intelligence	92

## Data Availability

The MIAS open access dataset used to support the findings of this study is included within the article.
